# An in-feed oral glucose test increases post-prandial high molecular weight adiponectin concentration in horses

**DOI:** 10.1093/jas/skag336

**Published:** 2026-10-12

**Authors:** Melody A de Laat, Fang I Li, Danielle M Fitzgerald, James M McGree

**Affiliations:** School of Biology and Environmental Science, Queensland University of Technology, Brisbane, Queensland, 4000, Australia; School of Biology and Environmental Science, Queensland University of Technology, Brisbane, Queensland, 4000, Australia; School of Animal Science, The University of Queensland, Gatton, Queensland, 4343, Australia; School of Mathematical Sciences, Queensland University of Technology, Brisbane, Queensland, 4000, Australia

**Keywords:** equine metabolic syndrome, hyperinsulinemia, laminitis, obesity, pony, total adiponectin

## Abstract

Adiponectin is a fat-derived adipocytokine that has been associated with laminitis risk in ponies. Factors that affect sampling timing for measurement of adiponectin concentration have been inadequately investigated. This study aimed to determine the effect of an oral glucose test (OGT) and fed-state on adiponectin concentration in horses and ponies. Blood samples were collected before and after an OGT in 85 horses and ponies, and high molecular weight (HMW) adiponectin concentration was measured. Total adiponectin concentration was measured in a subset of 25 animals. Assays validated for use with horse plasma were used. Next, HMW adiponectin was measured before and for up to 4 h after the consumption of three different diets in ponies: pasture (*n* = 16), a soyabean meal diet (*n* = 10), or diet with added maize and molasses (*n* = 5). The groups were compared with a Kruskal-Wallis rank sum test, with Dunn’s post-hoc test, and timepoints with a signed-rank, or Pearson’s, test. The ratios were compared with a paired t-test. Stepwise rank-based regression was used to consider multiple predictors for the change in HMW adiponectin concentration during the OGT. Friedman’s test was used to examine changes in concentration over time after meal consumption. Both the OGT and the diet containing maize caused a larger (*P* < 0.05) change in blood glucose concentration (0 h to 2 h) than the pasture or diet with soyabean meal. The OGT meal increased (*P* < 0.001) median [IQR] post-prandial HMW adiponectin concentration at the 2 h timepoint (4.02 [2.23-6.88] µg/mL), compared to the pre-test sample (2.75 [1.17-5.69] µg/mL). However, median (IQR) total adiponectin concentration did not change from the 0 h (7.78 [4.19-12.7] µg/mL) to the 2 h sample (6.48 [4.04-12.8] µg/mL). The proportion of HMW multimers in the total adiponectin concentration increased (*P* = 0.01) from 37% to 54% post-prandially. Pasture grazing and consumption of a soyabean or maize based meal did not result in a change in HMW adiponectin concentration over time. Blood samples for the measurement of HMW adiponectin should be taken before animals receive an in-feed OGT.

## Teaser

High molecular weight adiponectin was affected by the oral glucose test (OGT). Blood samples for the measurement of HMW adiponectin should not be taken in the immediate post-prandial period for animals receiving an OGT.

## Introduction

Hyperinsulinemia-associated laminitis (HAL) occurs when hyperinsulinemia causes weakening and damage to the lamellar structure of the horse’s hoof (Karikoski et al. 2015), resulting in lameness, loss of function, or euthanasia in unmanageable cases (Pollard et al. 2020). The disease is common, and early detection of the risk factors for HAL is important for risk management and disease prevention. However, HAL can be chronic and/or obscure, making it difficult to detect (Pollard et al. 2017). Thus, diagnosis of predisposing conditions, such as insulin dysregulation (ID), is valuable (Knowles et al. 2023). Measurement of insulin concentrations, including assessment of the pancreatic beta cell response to food and tissue insulin sensitivity, are all important aspects of characterizing ID in an individual animal (Durham et al. 2019). Other indicators for HAL risk can also be considered, and these can include a previous history of laminitis, concurrent diseases, management practices, and some adjunct biomarkers, such as measurement of adiponectin concentration (de Laat et al. 2019; Knowles et al. 2023).

Adiponectin is a fat-derived adipocytokine broadly associated with improving insulin sensitivity and reducing inflammation in humans (Turer and Scherer 2012), and in ponies low total adiponectin concentration has been associated with an increased risk of HAL (Knowles et al. 2023). Adiponectin circulates in three multimeric isoforms: low-, medium-, and high-molecular weight (HMW), and all contribute to the total adiponectin concentration (Schraw et al. 2008). Studies in humans measuring both total adiponectin, and the HMW isoform, have associated both total and HMW adiponectin concentrations with an insulin-sensitising effect (Blüher et al. 2007), while some studies found that the HMW multimer was a better indicator of biological activity (Basu et al. 2007). Both total and HMW adiponectin have been measured in horses and ponies and have been associated with various metabolic perturbations, including HAL risk (total) (Knowles et al. 2023) and obesity (total [Kearns et al. 2006] and HMW [Wooldridge et al. 2012]), with studies generally focusing on the relationships between adiponectin and metabolic parameters over the longer term (weeks to months).

Studies examining the short-term (hours) variability in adiponectin secretion and the factors that influence this are infrequent in horses. Given that measurement of adiponectin might be suggested as an additional test for Equine Metabolic Syndrome (Bertin et al. 2025) or HAL risk, factors that affect the circulating adiponectin concentrations in the short term are relevant to determining the appropriate time for sampling animals when adiponectin concentration is to be measured. Understanding factors that might change adiponectin concentration would enable a more informed approach to interpretation of adiponectin values in the context of obesity or HAL risk. This is particularly relevant to determination of laminitis risk, where temporary physiological increases or decreases in adiponectin concentration could lead to an incorrect estimation of disease risk from a point-in-time sample collection. A study found that total adiponectin concentration did not vary over a 24-h period in horses (Gordon and McKeever 2005), where hay (21.2% nonfibrous carbohydrate) and grain (38.2% nonfibrous carbohydrate) meals were provided, suggesting that short-term diurnal and meal-related fluctuations are minimal and unlikely to impact sampling timing and interpretation. However, another study (Meier et al. 2020) found that HMW adiponectin concentrations were increased within 4 h of feeding a meal high in non-structural carbohydrates (NSC; ∼12 g of NSC/kg bodyweight [BW]), indicating that the HMW multimer concentration might be more variable over the short term than total adiponectin concentrations.

One of the most common considerations when undertaking blood testing of animals for diagnostic purposes is fasted state. Horses are naturally trickle feeders (Boyd et al. 1988) and can be housed at pasture with free access to forage, so an understanding of the effect of meal consumption on HMW adiponectin concentration is relevant to the use of adiponectin as an adjunct biomarker for obesity or HAL. Further, the ability to combine tests, such as including measurement of adiponectin concentration when testing for ID would enable a more streamlined and affordable approach. However, commonly used tests for ID, such as the oral sugar (OST) and oral glucose tests (OGT), involve dosing the animal with sugar (Durham et al. 2019), and opportunities for sampling of animals might occur after test meal consumption has occurred. The primary aim of this study was to determine whether HMW and total adiponectin concentrations differ between the two sampling timepoints of the in-feed OGT in horses and ponies. A secondary aim was to determine the short-term effect of consumption of different meal types on HMW adiponectin concentrations in the immediate post-prandial period in ponies.

## Materials and methods

### Animals

This study comprised two parts, study one and study two, and made use of archived samples from 85 university-owned and privately owned horses and ponies. The main cohort was subsequently divided into four subgroups for further analyses (Figure 1). All studies from which the samples were sourced were approved by the Animal Ethics Committees of Queensland University of Technology and the University of Queensland (1600000825; 1800000196; 1900001168; 8480-18968) and carried out in accordance with the approval requirements. Consent forms were completed by animal owners and/or managers for the privately-owned animals. All animals were familiar with and remained in their environment and up-to-date with routine anti-parasitic programs and vaccinations relevant to the local area (Southeast Queensland, Australia). All animals were either mares or geldings (i.e. castrated males). They had received a clinical examination by a veterinarian (the first author) that included an assessment of body condition score (BCS; scale of 1–9) (Henneke et al. 1983) and cresty neck score (CNS; scale of 0–5) (Carter et al. 2009). All animals were weighed using an electronic scale (Active Scales, Mulgrave, Vic, Australia). The study exerted no control over the dietary or housing management of the animals in study one. A subset of the main population in study one was used to examine the total adiponectin concentration (subgroup 1; Figure 1). For study two, samples from different subset populations of the main cohort were used. These animals were university-owned and were separated into three groups (subgroups 2A, 2B, and 2C; Figure 1) based on their management (Table 1).

**Figure 1:**
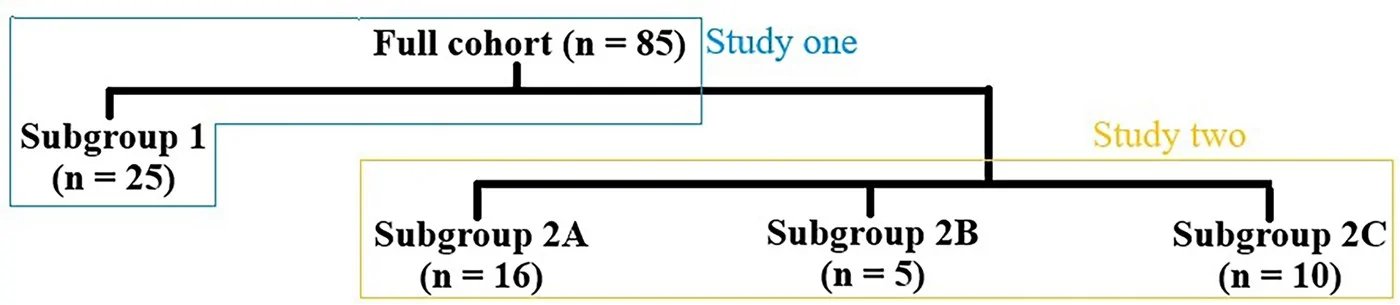
Flow chart of the design of a study of HMW adiponectin concentrations in a cohort of animals. Further investigations were undertaken in subgroups of the main population that included total adiponectin measurement (subgroup 1) and HMW adiponectin concentrations before and after feeding three different types of meals (subgroups 2A, 2B, 2C).

**Table 1:** Daily management regime for three groups of ponies in a meal response study where they were fed three different equine diets.

	Subgroup 2A	Subgroup 2B	Subgroup 2C
**Sample size**	16	5	10
**Diet type**	Pasture	Higher GR (H-GR)	Lower GR (L-GR)
**Turnout**	4 h at pasture	2–4 h in dirt lot	2–4 h in dirt lot
**Daily hay ration**	0.7% BW Rhodes grass hay (evening)	0.5% BW lucerne hay (midday) and 1% BWT lucerne hay (evening)	0.5% BWT lucerne hay (midday) and 1% BW Rhodes grass hay (evening)
**Morning forage**	Mixed species (Couch and Rye) at 8.83 g/kg BW (Fitzgerald et al. 2019b)	0.3% BW Lucerne chaff	0.25% BW Lucerne chaff
**Morning concentrate**	None	1.5g/kg BW maize^1^ and 1 g/kg soyabean^2^ meal	0.25% BW soyabean^2^ meal
**Molasses**	None	0.1 mL molasses^3^/kg BW	None
**V&M**	0.15 g/kg BW	0.15g/kg BW	0.2g/kg BW

Key: BW; bodyweight, GR; glycemic response, OGT; oral glucose test, V&M; Vitamin and mineral supplement (Gold Pellet, Kentucky Equine Research, KY, USA)

1 Micrmaize, Hygain, Vic, Australia.

2 Maxisoy, Energreen Nutrition, Qld, Australia.

3 Green Valley, Vic, Australia.

### Study design

In study one plasma samples collected during an OGT were used for measurement of HMW adiponectin, with a subset used to measure total adiponectin concentration. Animals had not been fed hay or a concentrate meal in the 3 h preceding the test but could have consumed a small amount of grass if any were present adjacent to, or in, their holding yard. All animals had received an in-feed OGT with the test meal containing a powdered dextrose dose of 0.75 g/kg BW that was mixed with 200 g wheat bran and ∼500 mL of water and combined with 0.3% BW lucerne chaff (de Laat et al. 2014). Miniature horses received only 100 g of wheat bran. This test relied on voluntary consumption of the test meal and >70% of the meal was eaten in all cases. The bucket was removed after 45 min, whether the animal has finished the meal or not. Samples were collected before (0 h) and 2 h after the meal was offered. An animal was considered negative for ID where the 0 h serum insulin concentration was ≤ 19 µIU/mL and the 2 h serum insulin concentration ≤ 84 µIU/mL with all other results classified as insulin-dysregulated (Durham et al. 2019).

In study two, the plasma samples were used for measurement of HMW adiponectin concentration in response to consumption of the animal’s usual morning food. The samples were collected before (0 h) and after (time course samples up to 4 h after access to the food) consumption of the meal. Study two included three different meal compositions that were intended to represent three types of meals that might be offered to horses and ponies that were sedentary or in light-to-moderate work. The meals elicited different glycemic responses (GR) in the animals to enable a comparison of pasture grazing with concentrate meals with (high glycemic response [H-GR]) and without (low glycemic response [L-GR]) added grain and molasses (Table 1). The animals had been acclimated to their diet for at least 2 wk before sampling. The samples for each meal type were collected on the same day, but the subgroups were sampled on different occasions. Samples were collected before turnout to pasture and then hourly for 4 h during pasture grazing in subgroup A, every 30 min for 2 h during and after consumption of the H-GR meal in subgroup B and every hour for 2 h during and after consumption of the L-GR meal in subgroup C. The nutritional content of each diet was analyzed at a National Authorities Testing Association accredited laboratory (Department of Primary Industries, NSW, Australia). Two different batches of the dietary components of the OGT test meal were tested to provide the nutritional content of the test meals. The NSC content of the diet was calculated according to the National Research Council method (NRC 2001).

### Sample processing and assays

Blood samples had been collected by jugular venipuncture in study one and from an indwelling venous catheter placed in a jugular vein under local anesthetic in study two. Aliquots (1 mL) of plasma derived from chilled ethylenediaminetetraacetic acid (Becton Dickinson, Macquarie Park, NSW, Australia) tubes that were processed and frozen within 30 min of collection and stored at −80 °C were used. Plasma HMW adiponectin concentrations were measured in the samples within 12 mo of collection (adiponectin is reported to be stable for years when stored at −80 °C [Rutkowski and Scherer 2014]) using a previously validated (Wooldridge et al. 2012; Fitzgerald et al. 2019a) ELISA (EZHMWAN-65K, Merck Millipore, Darmstadt, Germany) according to the kit protocol. Samples (*n *= 6) below the limit of detection (0.6 µg/mL) for horses were assigned a value of 0.3 µg/mL. Total adiponectin concentrations were analyzed with an immunoturbidimetric assay (51010, ImmunoDiagnostics, Sha Tin, Hong Kong) validated for use in horses (Menzies-Gow et al. 2019). Assay performance was acceptable for both assays. Blood glucose concentration had been measured at the time of sampling with whole blood using a validated (de Laat and Sillence 2023) glucometer (Accucheck-Go, Roche Diagnostics Australia). Serum had been derived from clot activator tubes (Becton Dickinson, Macquarie Park, NSW, Australia) left to clot for 30 min at room temperature before centrifugation, then removal of serum, which was immediately frozen. Serum insulin concentration was measured (Immulite 2000xPi, Siemens, Bayswater, Vic, Australia) at a commercial laboratory (VetPath, Perth, WA, Australia).

### Data analyses

Statistical analyses and graphical summaries were conducted using the R project for statistical computing and SigmaPlot (v.15, Systat Software Inc, San Jose, CA, USA). The accepted significance level was *P* ≤ 0.05, with *P* values < 0.1 considered for further evaluation during stepwise regression. Data are reported as median [IQR] or mean ± se. *A priori* power analysis (power = 0.8; α  =  0.05) was conducted for the main cohort to ensure sufficient sample size. For both studies, one and two, determination of the GR for each individual animal to their meal was undertaken by subtracting the 0 h (pre-prandial) blood glucose concentration from the 2 h post-prandial blood glucose concentration. Comparison of the GR to each meal type was achieved with a Kruskal-Wallis rank sum test, with Dunn’s post-hoc test, as the data were not normally distributed (Shapiro-Wilk test). The HMW and total adiponectin concentration data for the comparison between the 0 h and 2 h timepoints in study one did not follow a normal distribution according to the Shapiro-Wilk test, so they were analyzed with the Wilcoxon signed-rank test. Comparison of HMW-to-total adiponectin ratios was undertaken using a paired t-test. The insulin and blood glucose data were correlated with HMW adiponectin concentrations using Pearson’s test. A chi-squared test was used to compare the proportions of horses and ponies that had an increase in HMW adiponectin during the OGT. The remaining analyses for study one were performed using the difference between the concentrations at 2 h and 0 h (i.e. diff = 2 h–0 h). These data were either categorical or not normally distributed, so were initially analyzed with Mann-Whitney rank sum tests (horse/pony, ID status, and sex), a Kruskal-Wallis rank sum test (BCS and CNS), or correlated with Spearman’s test (age and BW) and reported as median [IQR]. Due to residuals not being normally distributed stepwise rank-based regression was used to consider multiple predictors for the change in HMW adiponectin concentration during the OGT together. Neither BCS nor CNS were included in the regression model due to small numbers in some ranks (Figure S1). In study two, Friedman’s test was used with Dunn’s test for post-hoc analyses to compare HMW adiponectin concentrations over time. The subgroups were compared for age, BW, and conditions scores with a Kruskal-Wallis test with Dunn’s post-hoc test for pairwise comparisons.

## Results

### Animals

The characteristics of the five groups of animals are described in Table 2. The cohort for study one contained horse and pony breeds, while in study two subgroups A, B, and C contained ponies and miniature horses. The groups were comparable in age, but comprised different breeds and varying body types, with BCS and CNS higher (*P* < 0.01) in subgroup A, than subgroup C (Table 2). Subgroups A, B, and C did not differ in BW (Table 2). All groups contained both normally insulin-regulated and insulin-dysregulated animals (Table 2).

**Table 2:** Characteristics of a cohort of horses and ponies used to examine the effect of eating on HMW and total adiponectin concentration, and subgroups of ponies within that cohort. Data are reported as median (IQR) and the subgroups were compared with Kruskal-Wallis test with Dunn’s post-hoc test.

	Whole cohort	Subgroup 1	Subgroup 2A	Subgroup 2B	Subgroup 2C	P-value
**Meal type**	OGT	OGT	Pasture	H-GR	L-GR	–
**Sex**	36 F; 49 M	11 M; 14 F	8 F; 8 M	4 F; 1 M	6 F; 4 M	–
**Type**	37 H; 48 P	10 H; 15 P	16 P	5 P	10 P	–
**ID**	48 N; 37 Y	14 N; 11 Y	8 N; 8 Y	2 N; 3 Y	6 N; 4 Y	–
**Age (years)**	15 [8.5–19.5]	9 [6.5–13.5]	11 [8–18]	12 [7–18]	10 [7–19]	0.61
**Bodyweight (kg)**	335 [163–515]	300 [122–612]	139 [120–162]	137 [123–148]	146 [108–303]	0.13
**BCS (1–9)**	5 [5–6]	5 [5–6]^a^	6 [5.25–7]^a,b^	6 [5.5–8.5]	5 [4.75–6]^b^	0.003
**CNS (0–5)**	2 [1–3]	2 [1–2.75]	2 [1.25–3]^a^	2 [1.5–3]	1 [0–2]^a^	0.03

Key: BCS; body condition score (Henneke et al. 1983), CNS; cresty neck score (Carter et al. 2009), ID: insulin dysregulated, F; Female, H; Horse, M; Castrated male, N; No, OGT; oral glucose test, P; Pony, Y; Yes, *; indicates the subgroup that was significantly different to the other subgroups.

### Dietary analyses and GR

For the OGT diet the NSC content of the batches tested was 1.58 g/kg BW and 1.39 g/kg BW “as-fed” for horses and ponies. This NSC content was slightly less for miniature ponies at 1.54 g/kg BW and 1.35 g/kg BW. The results of the nutritional analyses of the main meal components for the diets used for study two are provided in Table 3. The molasses was not analyzed for the purpose of the study, and the NSC content was taken to be the value stated by the manufacturer, which was 37.5 g/100g. The average intake rate for the pasture diet was reported previously (Fitzgerald et al. 2019b) and was used to estimate the “as-fed” NSC content of the pasture which was 0.63 g/kg BW NSC. The higher and lower GR diets provided 1.81 g/kg BW NSC and 0.62 g/kg BW NSC respectively (as-fed). The median [IQR] GR (i.e. 2 h minus 0 h blood glucose concentration) to the four meal types were different (*P* < 0.001), with higher responses to the OGT and H-GR meal (Figure 2).

**Figure 2:**
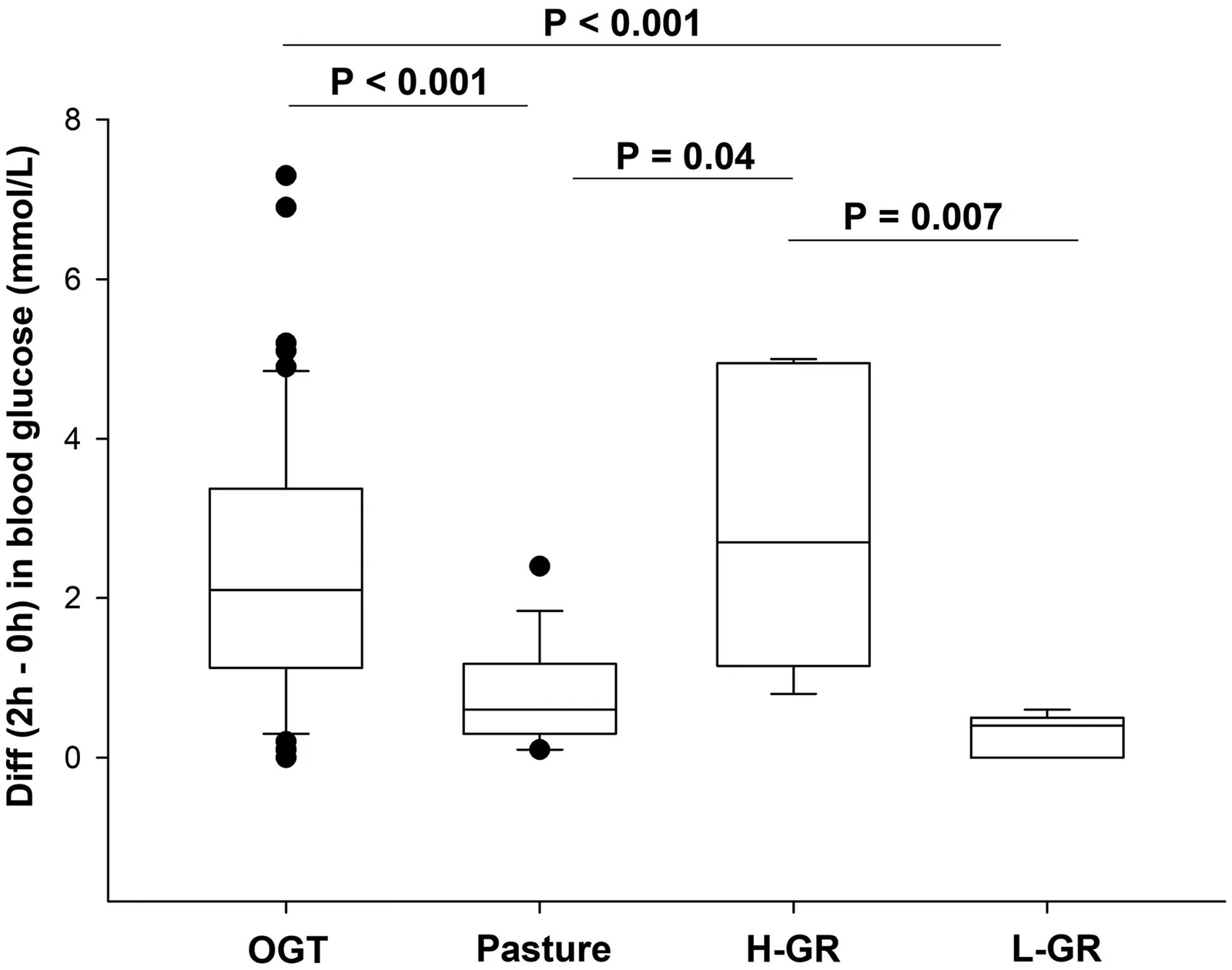
The difference in blood glucose concentration (mmol/L) after consumption of four meal types by ponies was compared. The oral glucose test (OGT; *n* = 85; 0.75 g/kg BW dextrose) and a high glycemic response (H-GR) concentrate diet with added grain and molasses (*n* = 5; 1.81 g/kg BW NSC as fed) elicited larger (*P* < 0.05) increases in blood glucose than the consumption of pasture (*n* = 16; 0.63 g/kg BW NSC as fed) or a low glycemic response (L-GR) concentrate meal without added grain or molasses (*n* = 10; 0.62 g/kg BW NSC as fed).

**Table 3:** Results of the nutritional analysis of the components of diets fed to three groups of ponies used in a study of HMW adiponectin concentration before and after feeding. Lucerne chaff H was used in the high energy diet and lucerne chaff L was used in the low energy diet. All variables were measured on a dry matter basis, except NSC which was calculated (NRC 2001).

	Lucerne chaff H	Lucerne chaff L	Pasture	Maize	Soyabean
**Dry matter (%)**	89	88.4	92.9	89.6	93
**ME (MJ/kg DM)**	10.5	9.8	8.1	13.1	10.1
**NSC (g/100g)**	24.7	18.5	7.1	80.9	–
**Crude protein (%)**	24.8	23.4	10	12.5	13.3
**Neutral detergent fiber (%)**	36	45	77	12	63

Key: DM; dry matter, ME; metabolizable energy, MJ; megajoule, NSC; non-structural carbohydrate, OGT; oral glucose test.

### Study one—oral glucose test

#### Adiponectin concentrations during the OGT

Across the full cohort, median HMW adiponectin concentration was 46% higher 2 h post-prandially than at baseline (0 h; *P* < 0.001; Figure 3A). However, 28/85 animals did not have an increase in HMW adiponectin concentration during the test. For animals with a positive change in HMW adiponectin concentration during the test (*n *= 57), neither the change in blood glucose (r = -0.06; *P* = 0.68) nor serum insulin (r = −0.23; *P* = 0.08) concentration was correlated with the increase in HMW adiponectin concentration. For the whole cohort, baseline and post-prandial concentrations were strongly correlated (r = 0.71; *P* < 0.001; Figure 3B). Baseline HMW adiponectin concentration was not correlated with baseline serum insulin (r = 0.008; *P* = 0.94) or blood glucose concentration (r = -0.02; *P* = 0.89). In contrast, post-prandial HMW adiponectin concentration was negatively correlated with both post-prandial serum insulin and blood glucose concentrations (*P* = 0.03; Figure S2).

**Figure 3:**
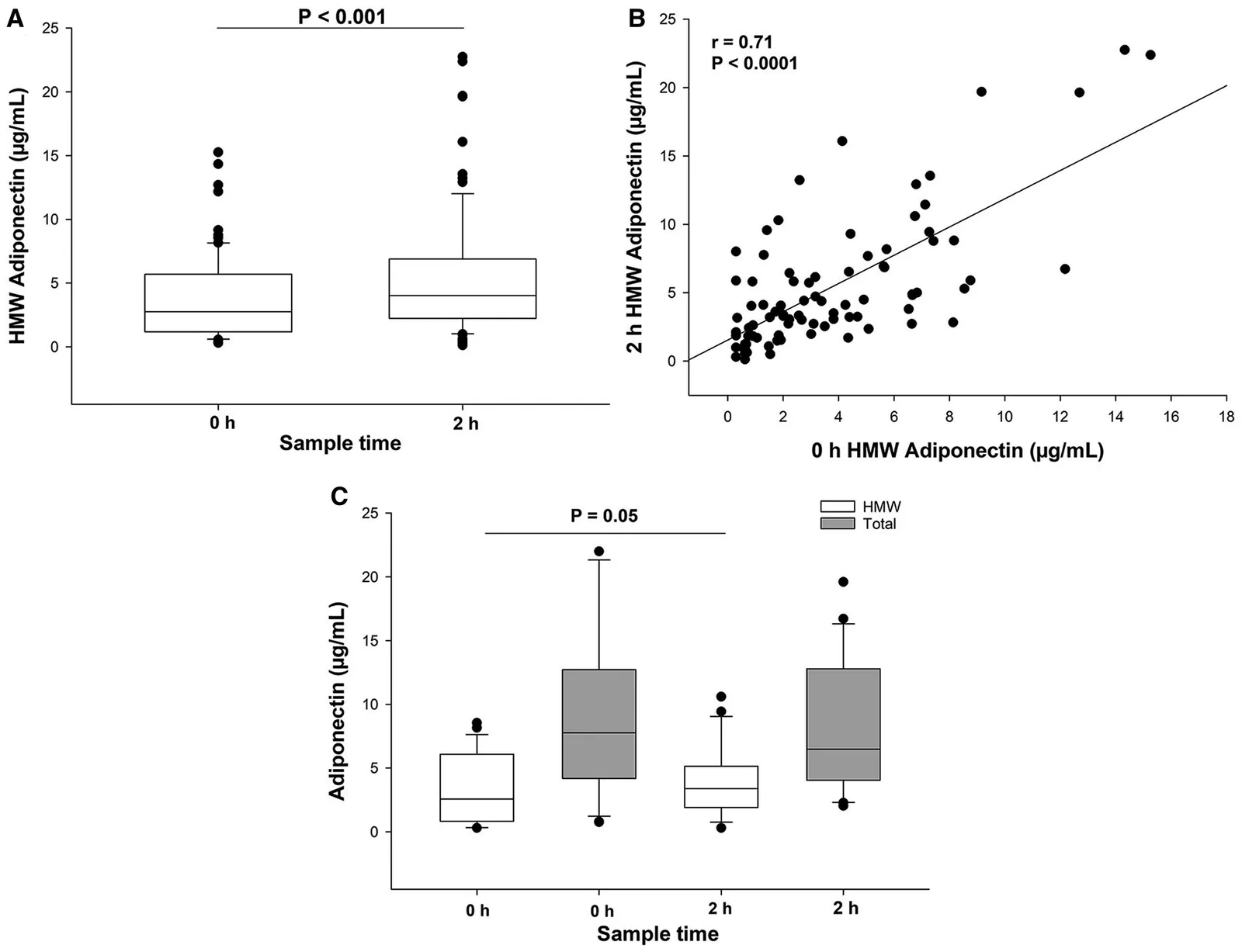
The median [IQR] HMW adiponectin concentration (µg/mL) was higher after eating an oral glucose test (OGT) meal, compared to before, in 85 horses and ponies (Panel A). The pre- and post-prandial HMW adiponectin concentrations were correlated during the OGT (Panel B). The total adiponectin concentration did not change during the OGT, but the ratio of the HMW-to-total increased (Panel C).

In subgroup 1 the total adiponectin concentration did not change (*P* = 0.21) from the baseline (0 h) to the post-prandial (2 h) sample (Figure 3C). However, the HMW adiponectin concentration increased (*P* = 0.05; by 19%) for this subgroup as it did for the main cohort (Figure 2C). The ratio of HMW-to-total adiponectin increased (*P* = 0.01) during the test with HMW adiponectin multimer comprising 37% of the total adiponectin concentration at the 0 h timepoint and 54% of the total at the 2 h timepoint.

#### Predictors of the difference in high molecular weight adiponectin

BW was negatively correlated (r = −0.33; *P* = 0.002) with the difference in HMW adiponectin concentration between the sampling timepoints, but age was not correlated (r = −0.07; *P* = 0.52). The difference in HMW adiponectin concentration during the OGT was higher (*P* = 0.04) in ponies than horses (Figure 4A), where 16/37 horses did not have a positive change in HMW adiponectin concentration during the OGT, compared to 12/48 ponies (*P* = 0.08). The difference in HMW adiponectin concentration was also higher in females (*P* = 0.002; Figure 4B) compared to castrated males. However, the difference in HMW adiponectin concentration was not affected by insulin regulation status when considered as a binary classification (*P* = 0.37; Figure 4C). Neither BCS nor CNS were correlated with the difference in HMW adiponectin concentration (Figure S1). Regression analysis found significant predictors of the difference in HMW adiponectin to be sex (*P* = 0.006) and BW (*P* = 0.004). Insulin regulation status (*P* = 0.06) was also considered due to the *P* value being < 0.1 and was included in further analyses to determine whether this *P* value would change when fewer variables were considered. In the final model, while all three variables were retained, only sex (*P* = 0.01) and BW (*P* = 0.006) remained as significant predictors after stepwise regression, with insulin regulation not associated (*P* = 0.11).

**Figure 4:**
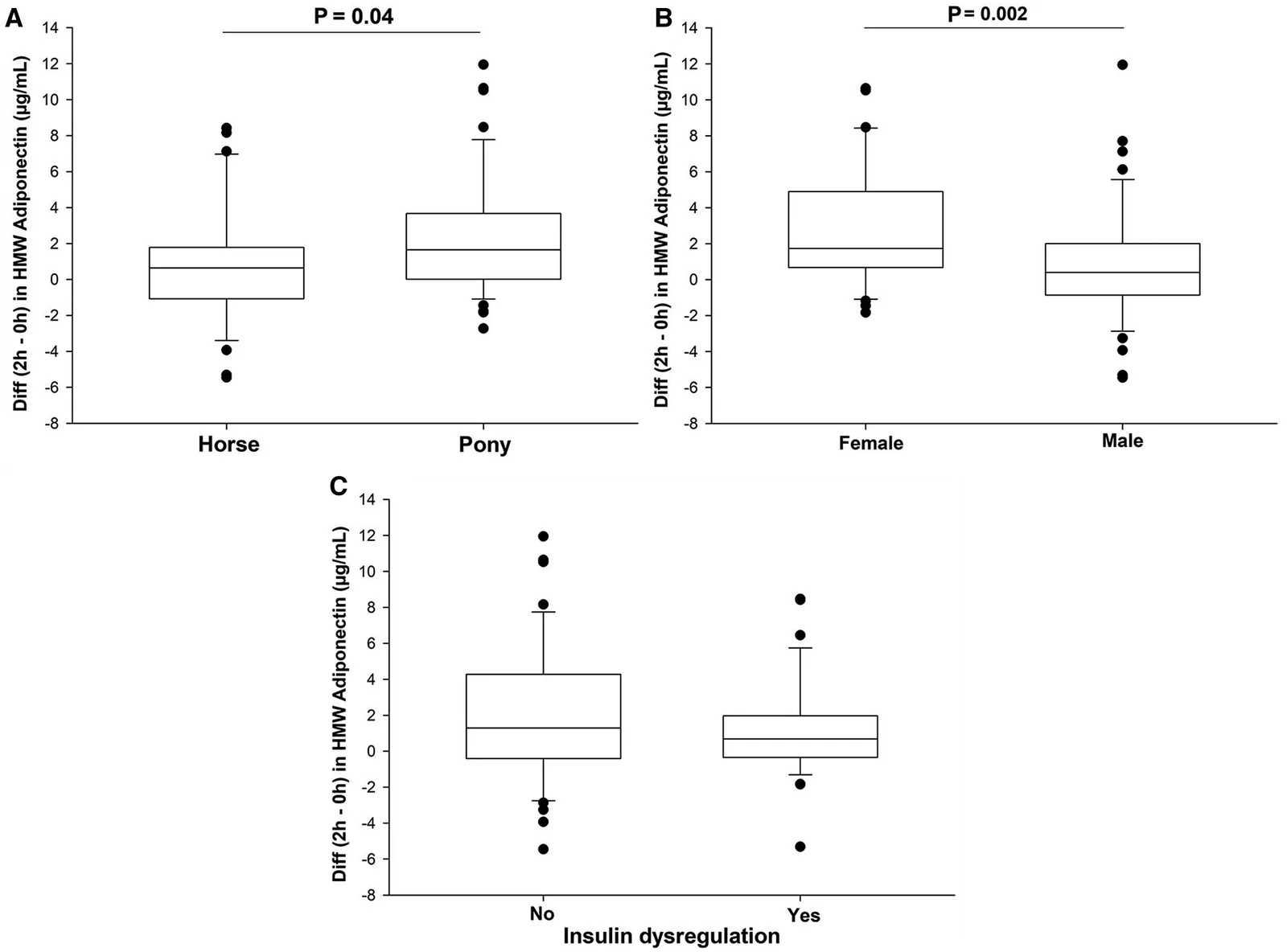
The difference in HMW adiponectin concentration (µg/mL) between 0 h and 2 h post-prandial sampling timepoints during an oral glucose test was different between horses and ponies (Panel A) and males and females (Panel B) but did not differ according to insulin regulation status (Panel C).

### Study two—meal responses

In subgroup A, the HMW adiponectin concentration did not change over the 4 h grazing time period (*P* = 0.46; Figure 5A). In subgroup B, the HMW adiponectin concentration did not change over the 2 h sampling time period during and after consumption of the H-GR meal (*P* = 0.076; Figure 5B). In subgroup C the HMW adiponectin concentration did not change over the 2 h sampling time period during and after consumption of the L-GR meal (*P* = 0.12; Figure 5C).

**Figure 5:**
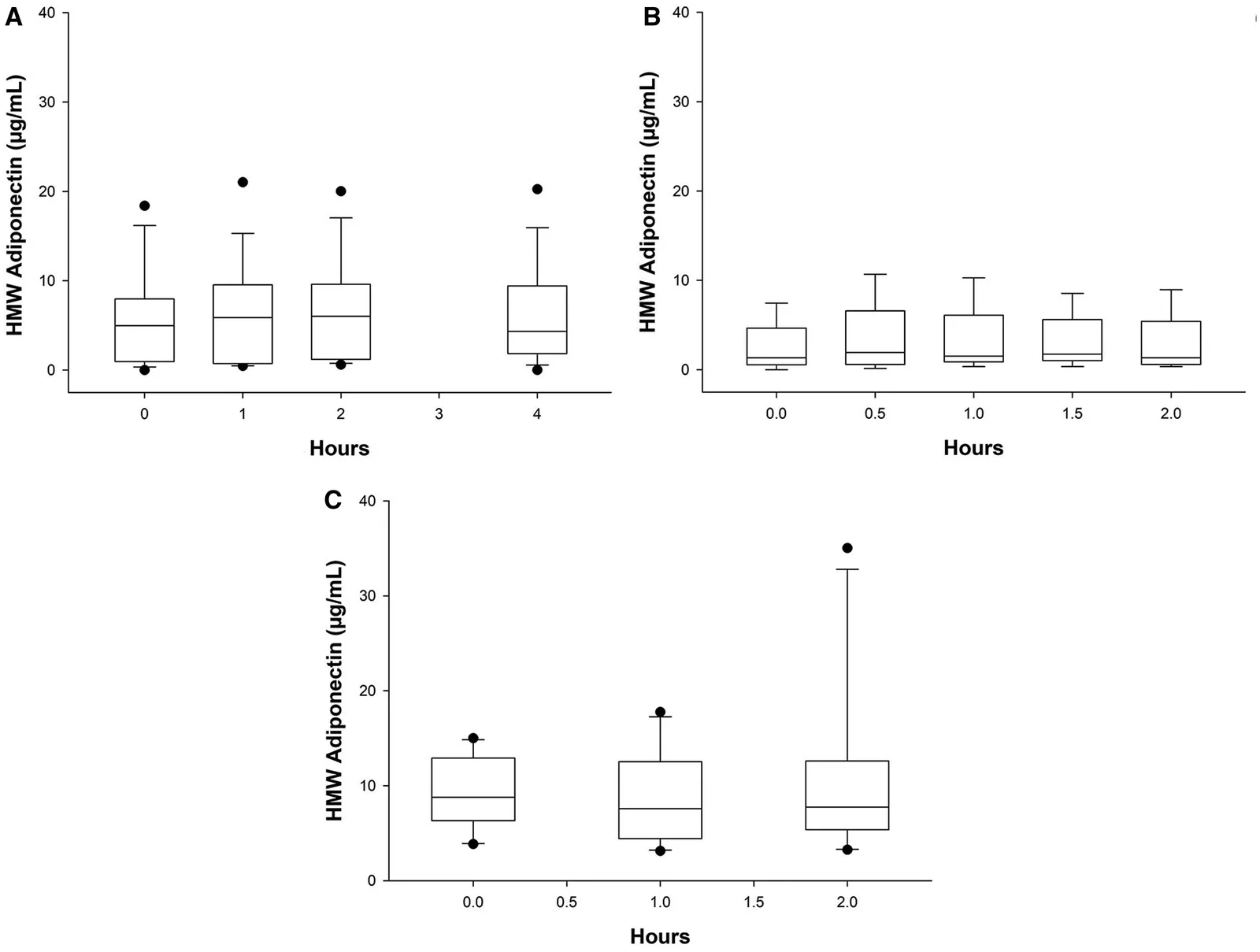
The HMW adiponectin concentration (µg/mL) did not change over a 4 h grazing time period (*n *= 16; Panel A). The HMW adiponectin concentration also did not change over the 2 h sampling time period during and after consumption of a high energy meal (*n *= 5; Panel B) or a low energy meal (*n *= 10; Panel C).

## Discussion

This study found that consumption of an OGT meal was associated with a 46% increase in HMW adiponectin concentration in horses and ponies. The mechanism for this increase at the 2 h sampling timepoint, compared to baseline concentration, is currently unknown. The data reported in human studies are conflicting. A decrease (6%–14%) in HMW adiponectin concentration at the 2 h sampling timepoint of an oral glucose tolerance test (using comparable glucose doses to the current study) has been reported in healthy individuals (Ozeki et al. 2009; Aso et al. 2013). In contrast, post-prandial increases in blood glucose concentration were positively associated with an increase in HMW adiponectin concentration after consumption of a liquid carbohydrate test meal in humans with diabetes (Iwase et al. 2009), which suggests that metabolic disease might influence HMW adiponectin responses during an oral glucose challenge. However, insulin regulation status was not a predicting factor for the change in HMW adiponectin during the OGT in this study. Administration of adiponectin reduces circulating glucose in humans (Combs et al. 2001), suggesting that a compensatory response to the oral dextrose intake was possible here. In addition, studies that have employed hyperinsulinemic and hyperglycemic clamp techniques in human subjects found a suppressive effect of insulin on adiponectin secretion that could be prevented with concurrent glucose infusion (Blümer et al. 2008). Both insulin and glucose concentrations increase during an OGT in horses (de Laat et al. 2016), and the absolute values of insulin and glucose were associated with HMW adiponectin in the post-prandial samples in the current study, supporting some correlation between these variables in individual animals, as shown previously (Frank and Walsh 2017; Knowles et al. 2024). However, the magnitude of the increase in insulin and glucose during the test was not associated with the size of the increase in HMW adiponectin, which does not suggest that a simple proportional relationship between them exists during an OGT. Taken together, these data suggest that further investigation of the interactions between glucose, insulin, and HMW adiponectin during an OGT could help to elucidate the mechanisms by which HMW adiponectin concentration increased during the test in the current study.

Mechanisms other than those directly associated with insulin and glucose regulation are possible. Acute hyperglycemia in humans has been shown to increase the release of pro-inflammatory cytokines (Esposito et al. 2002; Peiró et al. 2016). Adiponectin has anti-inflammatory properties (Tilg and Moschen 2006), and perhaps the increased HMW adiponectin seen here was a counter-regulatory response to an inflammatory response. Alternatively, the clearance rate of HMW adiponectin (∼80 min) has been shown to be longer than that of the LMW isoforms (30–55 min) in healthy rodents, suggesting that slower clearance might have contributed to the increased concentrations detected (Halberg et al. 2009). However, clearance rates for adiponectin have not been determined specifically for horses.

Total adiponectin concentration was not affected by the OGT meal, albeit this conclusion was based on data from a smaller number of animals compared to the HMW adiponectin dataset. To the authors’ knowledge, total adiponectin concentration has not previously been measured in the postprandial sample of an OGT or OST and is often only measured in the baseline sample (Frank and Walsh 2017; Barnabé et al. 2026), so the effect of the test on total adiponectin concentration has not been previously demonstrated. However, a study has reported that total adiponectin concentration was unchanged by an oral grain test (Gordon and McKeever 2006), which is consistent with the current result. In the human studies discussed previously where HMW adiponectin was reduced during an oral glucose tolerance test (Ozeki et al. 2009; Aso et al. 2013), the total adiponectin concentration remained unchanged. This is consistent with a separate study on human patients where total adiponectin concentrations were unchanged during an oral glucose tolerance test in human patients with a healthy BW but decreased over the 2 h test in obese patients (Larsen et al. 2019). Given that measurement of total adiponectin concentration is commercially available for horses in some geographical locations, further investigation of total adiponectin concentrations during an OGT using a larger range of horse and pony breeds, in the regions where the test is available, would be beneficial.

An important finding of the current study was that the HMW fraction increased as a proportion of the total concentration during the OGT. Studies in rodents and humans have shown that adiponectin isoforms do not change structure once in the blood (Schraw et al. 2008), indicating that a direct effect on multimer assembly in the adipocyte was more likely. Further, the intracellular assembly of HMW multimers of adiponectin requires glycosylation of lysine residues, and incubation of adipose tissue explants from humans with supraphysiological glucose coincided with increased formation of HMW multimers (Richards et al. 2006), which could provide support for a potential effect of oral glucose on enhanced HMW multimer assembly in the current study. The reason for the change in the ratio of multimers to hexamers and trimers is unknown, but in other species increased production of the HMW multimer has been associated with improved glucose disposal in adipocytes (Pandey et al. 2019) and myocytes (Yamauchi et al. 2002), suggesting that increased HMW production could have occurred to help counteract the glucose load. The proportion of HMW multimers in the baseline samples of 37% was similar to a previous report in lean horses, where HMW isoforms comprised 32% of the sample (Wooldridge et al. 2012), noting that the lack of equine-specific assays for adiponectin could contribute to variation as could the need to compare HMW and total concentrations that were measured with different assays. These proportions are lower than reported for mice (∼65%) and humans (∼55%–60%) (Lara-Castro et al. 2006; Schraw et al. 2008). The proportion of HMW adiponectin in an individual’s total adiponectin concentration has been shown to be an indicator of metabolic health in humans (Lara-Castro et al. 2006), but no studies of this nature have been undertaken in horses.

There was a larger change in HMW adiponectin during the OGT in ponies, compared to horses, and an accompanying effect of BW. A recent study showed that HMW adiponectin concentrations were higher in horses, than ponies (de Laat et al. 2026). Some pony breeds are known to be less insulin-sensitive than horse breeds (Coffman and Colles 1983), and a more marked response to the OGT test meal could have been associated with differences in glucose and insulin regulation during the test. Taken together these data suggest that the regulation of adiponectin secretion or action might inherently differ between horses and ponies, as insulin sensitivity can, and increasing our understanding of what underpins these differences is necessary. The insulin regulation status of the individuals in this study did not seem to play a significant role in modulating the response to the test, so investigation of other reasons for the differences between horses and ponies, such as potential genetic differences, is required. The reason for the larger change in HMW adiponectin concentration in mares, compared to geldings, during the OGT is unclear. It has been shown that testosterone has a suppressive effect on adiponectin production in humans (Böttner et al. 2004; Ohman-Hanson et al. 2016), which was attributed to differences in the HMW multimer in a study using genetically-modified, adiponectin deficient mice (Schraw et al. 2008). However, as all male horses in this study were castrated, the exact mechanisms for the difference found in this study remain unknown.

The OGT and OST are commonly undertaken during diagnostic work-up of a horse or pony suspected of being insulin-dysregulated (Smith et al. 2016; Bertin and de Laat 2017). As horses and ponies with ID are also at an increased risk of HAL, taking a sample for measurement of adiponectin concentration at the same time as an OGT/OST might be considered in these animals (Bertin et al. 2025). While the HMW adiponectin multimer is currently measured in research settings, total adiponectin concentration is available in some countries for clinical use. Based on the results of study one, samples for the measurement of HMW adiponectin should be taken in the fasted or resting state, prior to an OGT being performed, while sampling timing for total adiponectin concentration might be less important. Whether an increase in HMW adiponectin concentration also occurs after the per os dose of corn or glucose syrup administered during an OST requires investigation. The OST does not include provision of a meal and can be undertaken in either a low- or high-dose format (Jocelyn et al. 2018), both of which use a lower glucose dose than is provided during the in-feed OGT (Durham et al. 2019). It is unknown how the more rapid absorption of the lower glucose dose in the OST would affect HMW adiponectin concentration.

To further investigate the nature of the response to the OGT meal, study two was undertaken, but we found no effect of the meals on HMW adiponectin concentration. Previously, HMW adiponectin concentration was found to increase after eating a high-energy diet in a study of a large number of ponies (Meier et al. 2020), so further investigation of the effect of meals high in NSCs is warranted given the small sample size available for analysis here. In comparison, it has been shown in horses that diets supplemented with fat or glucose did not result in a change in HMW adiponectin concentration, compared to a control group, after a 20-wk feeding period (Bamford et al. 2016). In addition, a study of ponies grazing pasture over 22 wk found that plasma total adiponectin concentration declined, potentially in association with increased body condition/obesity (Barnabé et al. 2026). Taken together, these data show that any short-term responses to diet composition might not be consistent with longer-term dietary changes that are more likely to result from physiological changes related to metabolic status, such as body condition and insulin regulation. Adaptation to dietary nutrients is an important regulator of adiponectin secretion over the longer term (Janiszewska et al. 2021), and long-term regulation is quite distinct from the short-term response studies reported here.

It is important to note that pasture can be highly variable in NSC content, and the current finding that pasture grazing did not result in short-term changes in adiponectin concentration cannot be extrapolated to all types of pasture. As such, it is feasible that grazing certain types of pasture could cause an increase in HMW adiponectin as the OGT did. Overall, this study found that sampling for adiponectin concentration is best performed at the baseline (0 h) timepoint of an OGT or before a meal with a high NSC content to ensure any short-term responses to eating that might be further compounded by sex, BW or type, are avoided. The short-term increases in HMW adiponectin shown here seemed are potentially associated with the glucose or NSC content of the diet provided, and further study on the relationship between post-prandial hyperglycemia and adiponectin secretion in horses is warranted.

## Supplementary Material

skag336_Supplementary_Data

## Data Availability

The data underlying this article will be shared on reasonable request to the corresponding author.

## Abbreviations

BCS: body condition score
BW: bodyweight
CNS: cresty neck score
EMS: equine metabolic syndrome
GR: glycemic response
HAL: hyperinsulinemia-associated laminitis
HMW: high molecular weight
H-GR: high glycemic response
ID: insulin dysregulation
L-GR: low glycemic response
NSC: non-structural carbohydrate
OGT: oral glucose test
OST: oral sugar test
